# Prior metabolite extraction fully preserves RNAseq quality and enables integrative multi-‘omics analysis of the liver metabolic response to viral infection

**DOI:** 10.1080/15476286.2023.2204586

**Published:** 2023-04-24

**Authors:** Zachary B Madaj, Michael S. Dahabieh, Vijayvardhan Kamalumpundi, Brejnev Muhire, J. Pettinga, Rebecca A. Siwicki, Abigail E. Ellis, Christine Isaguirre, Martha L. Escobar Galvis, Lisa DeCamp, Russell G. Jones, Scott A. Givan, Marie Adams, Ryan D. Sheldon

**Affiliations:** a Bioinformatics and Biostatistics Core, Van Andel Institute, Grand Rapids, MI, USA; b Core Technologies and Services, Van Andel Institute, Grand Rapids, MI, USA; c Department of Metabolic and Nutritional Programming, Van Andel Institute, Grand Rapids, MI, USA; d Mass Spectrometry Core, Van Andel Institute, Grand Rapids, MI, USA; e Genomics Core, Van Andel Institute, Grand Rapids, MI, USA

**Keywords:** Transcriptomics, metabolomics, systems biology, integrated omics, mass spectrometry, RNA

## Abstract

Here, we provide an in-depth analysis of the usefulness of single-sample metabolite/RNA extraction for multi-‘omics readout. Using pulverized frozen livers of mice injected with lymphocytic choriomeningitis virus (LCMV) or vehicle (Veh), we isolated RNA prior (RNA) or following metabolite extraction (MetRNA). RNA sequencing (RNAseq) data were evaluated for differential expression analysis and dispersion, and differential metabolite abundance was determined. Both RNA and MetRNA clustered together by principal component analysis, indicating that inter-individual differences were the largest source of variance. Over 85% of LCMV *versus* Veh differentially expressed genes were shared between extraction methods, with the remaining 15% evenly and randomly divided between groups. Differentially expressed genes unique to the extraction method were attributed to randomness around the 0.05 FDR cut-off and stochastic changes in variance and mean expression. In addition, analysis using the mean absolute difference showed no difference in the dispersion of transcripts between extraction methods. Altogether, our data show that prior metabolite extraction preserves RNAseq data quality, which enables us to confidently perform integrated pathway enrichment analysis on metabolomics and RNAseq data from a single sample. This analysis revealed pyrimidine metabolism as the most LCMV-impacted pathway. Combined analysis of genes and metabolites in the pathway exposed a pattern in the degradation of pyrimidine nucleotides leading to uracil generation. In support of this, uracil was among the most differentially abundant metabolites in serum upon LCMV infection. Our data suggest that hepatic uracil export is a novel phenotypic feature of acute infection and highlight the usefulness of our integrated single-sample multi-‘omics approach.

## Introduction

The metabolome is an incredibly diverse collection of small molecules (<1,500 Da) in biological systems involved in virtually every cellular process, including cellular energy production, macromolecule synthesis, epigenetic modifications, cell signalling and more (for recent reviews see [1–6]). It responds rapidly (in seconds) to both internal (signalling, allostery) and external (nutrient availability, environment) stimuli [7–11] and, as a result, is regarded as the most proximal manifestation of phenotype [12–15]. Indeed, altered metabolism is a hallmark feature of many human diseases, such as cancer [16–18], neurodegenerative disease [19–21] and infection [22–25]. For these reasons, metabolomics has emerged as an essential approach when interrogating metabolic phenotypes and has proven indispensable for understanding physiological and pathological mechanisms (reviewed in [26]).

While metabolomic profiling is useful in identifying metabolites or metabolic pathways involved in a phenotype, it fails to capture metabolic dynamics or directionality and can be difficult to interpret in isolation [27]. Conversely, transcriptomic approaches, such as RNA sequencing (RNAseq), provide a semi-quantitative view of transcript abundance that gives insight into how a cell is responding to an ongoing insult or stimulus through up- and down-regulation of gene expression. However, in the context of metabolism, neither the expression of a gene nor its translation to a protein necessarily equates to the activity of a given metabolic pathway [28], and therefore, RNAseq is ill-suited to solely define metabolic phenotype. To this end, combining the orthogonal readouts of metabolomics and RNAseq can help paint a more complete picture of metabolic phenotype [12].

Both RNAseq and mass-spectrometry-based metabolomics rely on the destruction of the sample from which the target molecules are isolated. The sample must be regenerated or sampled multiple times for additional analyses, introducing sampling errors and limiting feasibility in low-quantity or rare sample types. For metabolomics, the wide range of compound chemistries present in the metabolome poses a particular challenge as no extraction method is suitable for the entire metabolome. A widely used approach to address this problem is the homogenization of the sample with ice-cold 80% methanol, which both quenches metabolism through protein precipitation and extracts polar and semi-polar metabolites involved in central carbon metabolism [29,30]. After extraction, the insoluble fraction contains protein, neutral lipids and nucleic acids. Numerous studies have demonstrated that the isolation of these molecules for further analyses is possible with additional processing steps [31–37]. However, these studies do not compare the quality of these other target compounds downstream of metabolite extraction *versus* a traditionally extracted control. As such, it is unknown whether this additional processing influences the downstream ‘omics readout and ultimately the biological interpretation.

To address this gap, the main objective of this study was to determine the effects of prior metabolite extraction on RNAseq using liver tissue from mice infected with a model of acute lymphocytic choriomeningitis virus (LCMV) infection. The liver is both a master regulator of systemic metabolic homoeostasis and an immunological hub owing to its large population of non-parenchymal cells including phagocytes (Kupffer cells, macrophages, dendritic cells, neutrophils) and lymphocytes (innate lymphoid cells, T cells and B cells; reviewed in [38]). LCMV is commonly used to study adaptive immunity as it induces strong systemic effects in many organs, including spleen, brain, kidney and liver in mice [39]. At the peak of LCMV infection, although the liver bears the second largest viral burden (next to the spleen), it possesses the capacity to clear the infection, while robust viral titres are still observed in brain and kidneys [39]. Thus, the LCMV-infected liver (*versus* non-infected controls) is an excellent model system that features strong metabolic and transcriptional responses, providing rich biological contrast, and that is large enough to be sampled multiple times to allow for direct comparison of extraction methods. Our results demonstrate that prior metabolite extraction does not impact RNAseq quality. We further use integrated single-sample metabolomics and transcriptomics data that support a novel role for pyrimidine metabolism in the hepatic response to LCMV infection.

## Material and methods

### Animal Care/Mice

Twenty male-C57/BL6 mice were selected for the study. All mice were maintained in a climate-controlled environment on a 12 h:12 h light–dark cycle with *ad libitum* access to water and a standard chow diet. At 11 weeks of age, mice were randomly selected to receive an intraperitoneal injection of lymphocytic choriomeningitis virus (LCMV) (Armstrong strain, 2 × 10^5^ PFU by intravenous injection) or PBS vehicle (Veh) [40]. 2.5 days post-infection, mice were anaesthetized with an isoflurane vaporizer. When anaesthetized, the liver was removed and snap-frozen in liquid N_2_ for later processing. All animal protocols were approved by the Van Andel Institute’s Institutional Animal Care and Use Committee.

### Liver processing

Frozen livers were pulverized into a fine powder using a mortar and pestle pre-chilled in liquid N_2_. Being careful to avoid thawing of powdered liver, two roughly 30 mg aliquots of each liver were rapidly weighed into liquid N_2_ chilled tubes. An aliquot was weighed into a liquid N_2_ chilled 1.5 mL Eppendorf tube for later RNA extraction. The other aliquot was weighed in to a 2.0 mL Omni Bead Ruptor (19–627, Omni) tube for metabolite extraction. Precise weights were then recorded, and samples were stored at −80°C until processing.

### Metabolite extraction

Metabolites from the liver and plasma were extracted with 80% methanol (v/v). For liver, frozen tissue was pulverized with a mortar and pestle under liquid N_2_. Aliquots of 30–40 mg were prepared by weighing rapidly to avoid thawing. Samples were homogenized in a bead mill homogenizer with a liquid N_2_ chilled homogenization chamber to prevent sample warming. Extraction solvent volume was adjusted for each sample to achieve a fixed 40 mg tissue/mL extraction solvent ratio. For plasma, 20 µL of plasma was extracted in 980 µL of 80% MeOH (v/v). Both liver and plasma samples were incubated on wet ice for 60 min to ensure complete metabolite extraction and precipitation of protein and nucleic acids. Extracts were then centrifuged at 17,000 × g for 10 min at 4°C. The supernatant was transferred to a fresh 1.5 mL Eppendorf tube, dried in a Speedvac (Genevac) and then stored at −80°C for later LC/MS analysis. The insoluble pellet from liver extracts was dried and stored at −80°C for subsequent RNA extraction.

### Metabolomics

Metabolite profiling analysis was completed using ion-paired reversed phase liquid chromatography using an HPLC (1290 Infinity II, Agilent Technologies, Santa Clara, CA) coupled to a triple quadrupole mass spectrometer (6470, Agilent) with electrospray ionization operated in negative mode. The column was a ZORBAX Rapid Resolution HD (2.1 × 150 mm, 1.8 µm pore size; 759700–902, Agilent). Mobile phase A was 3% methanol (in H_2_O), and mobile phase B was 100% methanol. Both mobile phases contained 10 mM of the ion-pairing agent tributylamine (90780, SigmaAldrich, St Louis, MO, USA), 15 mM acetic acid and 2.5 µM medronic acid (5191–4506, Agilent Technologies, Santa Clara, CA, USA). The LC gradient was as follows: 0–2.5 min 100% A, 2.5–7.5 min ramp to 80% A, 7.5–13 min ramp to 55% A, 13–20 min ramp to 99% B, 20–24 min hold at 99% B. Flow rate was 0.25 mL/min, and the column compartment was heated to 35°C. The column was then backflushed with 100% acetonitrile for 4 min (ramp from 0.25 to 0.8 mL/min in 1.5 min) and re-equilibrated with mobile phase A for 5 min at 0.4 mL/min. Full LC-MS parameters and measured metabolite peak areas are available in **Supplemental File 1**.

Differential abundance of metabolites collected from liver tissue and blood serum was analysed separately using R v 4.1.0 via a workflow, inspired by the R package DEP (https://bioconductor.org/packages/release/bioc/html/DEP.html). To start, metabolites with zero variance were filtered out, and then the remaining metabolites were assessed for missingness. Metabolites with missing data were determined to be not-missing-at-random as these metabolites had lower overall mean abundance compared to metabolites without any missingness. To address this potential source of bias, metabolites with less than 30% missingness were imputed from a left truncated distribution with parameters estimated using quantile regression (https://cran.rstudio.com/web/packages/imputeLCMD/index.html). Metabolite data were then log2 transformed, and a variance stabilizing normalization was applied to prepare data for analysis via LIMMA ebayes; both the Limma linear regression and empirical Bayes were fit using robust methods to prevent individual animals and/or metabolites from exerting excessive influence on estimates (https://bioconductor.org/packages/release/bioc/html/limma.html). Batch was included as covariate in the LIMMA linear models. Hypothesis tests were then multiple-testing adjusted via Benjamini-Hochberg to maintain a 5% false discovery rate (FDR).

### RNA Extraction and Quality Control

RNA was isolated directly from frozen liver tissue (RNA) or from post-metabolite extracted liver tissue (MetRNA). Samples were pulverized using QIAzol Lysis Reagent and a Tissue Lyser II. RNA was then purified from the lysates using RNeasy Plus Universal Mini Kit column-based isolation per manufacturer’s instructions. Isolated RNA was quantified by Promega Quantus and measured for integrity using Agilent Bioanalyzer. We used the calculated RNA Integrity number (RIN), which used the entire electrophoretic trace of RNA sample (including degradation products) to compare the RNA integrity between our samples. Differences in unique mapping rates between LCMV vs Veh and MetRNA vs RNA were tested using beta regression via the R package ‘betareg’ (https://cran.r-project.org/web/packages/betareg/index.html).

### RNA Sequencing

Following quality control, stranded RNA libraries were prepared from 500 ng RNA using the KAPA – mRNA Hyper Prep (08098140702, Roche). RNA libraries were then normalized, pooled and sequenced on Illumina NovaSeq S1 flow cell using paired end, 50 base pair reads to an average depth of 50 M reads per sample. Base calling was performed by Illumina RTA3, and the output of NCS was demultiplexed and converted to FastQ format with Illumina Bcl2fastq v1.9.0.

Adapters and low-quality bases (Phred >30) were trimmed from raw reads using Trim_Galore (https://www.bioinformatics.babraham.ac.uk/projects/trim_galore/). These trimmed reads were then aligned to the GRCm38 reference genome using the STAR aligner v2.7 (https://pubmed.ncbi.nlm.nih.gov/23104886/). Aligned read counts were imported into edgeR (https://doi.org/10.1093/bioinformatics/btp616) wherein library sizes were normalized using the trimmed mean of *M* values method and pairwise comparisons for differential expression were performed using the quasi-likelihood approach as in (). Raw and processed data are available under the GEO Accession ID GSE226930.

### Power

The current experimental design (*n* = 10/group) enabled an expected 95% power to detect all true fold-changes >1.5 and >99% power for all fold-changes >1.75 at a read depth of 40 M and a conservative true null rate of 99.9%.

### Statistical analysis of RNA dispersion

To determine if isolating RNA and metabolites from the same sample altered variance profiles within the RNAseq gene expression estimates (e.g. does coextraction introduce additional noise into the measurements), we compared the mean absolute difference between samples where only RNA was isolated and samples where RNA was isolated post-metabolite extraction, stratifying them by experimental group (LCMV/Veh) (https://cran.r-project.org/web/packages/Hmisc/index.html). The mean absolute difference was chosen as a measure of dispersion because it is non-parametric and has a natural interpretation. The distributions of the fold-change in dispersion were then assessed for skewness, which could indicate a biased change in dispersion related to the extraction method, particularly if this bias is conserved over both LCMV and vehicle groups. Finally, the mean absolute differences from the two extraction methods were correlated using Pearson’s rho.

### Pathway enrichment analysis & multi-omics

Multi-omic pathway enrichments were performed using MetaboAnalyst’s v5.0-‘joint-pathway analysis’ where we investigated which metabolic pathways were most impacted by LCMV infection (https://www.metaboanalyst.ca/). All differentially expressed genes, along with their fold-changes, were entered as gene symbols, and all differentially abundant metabolites were entered as their compound names. Metabolite fold-changes were not included to avoid the enrichment being dominated by large fold-changes as small metabolic differences can have large impacts. Pathway impact and Benjamini-Hochberg FDR adjusted significance were estimated using hypergeometric tests and MetaboAnalyst’s ‘tight integration’ method [41], which combines queries into one pooled universe. The ‘betweenness centrality’ was emphasized to identify pathways where the flow of information was heavily impacted. The ‘overall weighting’ and ‘pathway-level’ weighting methods were also explored in combination with each centrality measure (degree, betweenness and closeness), resulting in six joint-enrichments. MetaboAnalyst was also used to examine individual impacts of DEGs and DAMs on all pathways and metabolic pathways, respectively (options in MetaboAnalyst: ‘Metabolic pathways (metabolite only)’ and ‘All pathways (gene only)’). The top five impacted pathways are reported. A more common gene set enrichment analysis via the R package clusterProfilier [42] and Reactome pathways were also conducted for comparison.

## Results

### RNA quality

First, we assessed whether there were any overt differences in RNA quality from prior metabolite extraction. Both methods yielded RIN scores >9 and a 260/280 ratio of ~2 (Figure S1A-B). MetRNA had an increased 260/230 ratio (Figure S1D), which could be an indication of removal of contaminants, and improved RNA yield (Figure S1C). Thus, the metabolite extraction carried out prior to RNA purification does not compromise RNA quality and might serve to improve contaminate removal and RNA yield.

### RNAseq analysis

RNAseq was performed on both RNA and MetRNA samples. Unique mapping rates, i.e. the number of sequenced fragments that map to a unique location in the genome, averaged 75% for vehicle and 82% for LCMV-treated samples (Figure S1E). The increased unique mapping rate in LCMV over vehicle was hypothesized to be due to gene activation in response to viral infection, as LCMV had more unique gene features after trimming (Figure S1F). We further observed a small but statistically significant increase in the unique mapping rate of MetRNA vs RNA, which correlates to the improved 260/230 ratio. No additional genes were detected as a result of this small difference in unique mapping rate. Samples were diverse and expressed 15,535 unique gene features after trimming (Figure S1F). Within individual animals, absolute transcript abundance was tightly correlated between MetRNA and RNA (Figure S2A). Gene expression within the RNA samples explained >98% of variance in the MetRNA samples for each animal. Furthermore, after trimming, there was ~ 96% overlap in genes detected in each sample between the two extraction methods (Figure S2B). There were no genes specific to only one extraction method, that is, if a gene had measured counts in one extraction method it was also measured in at least a subset of animals in the other.

**Figure 1.**
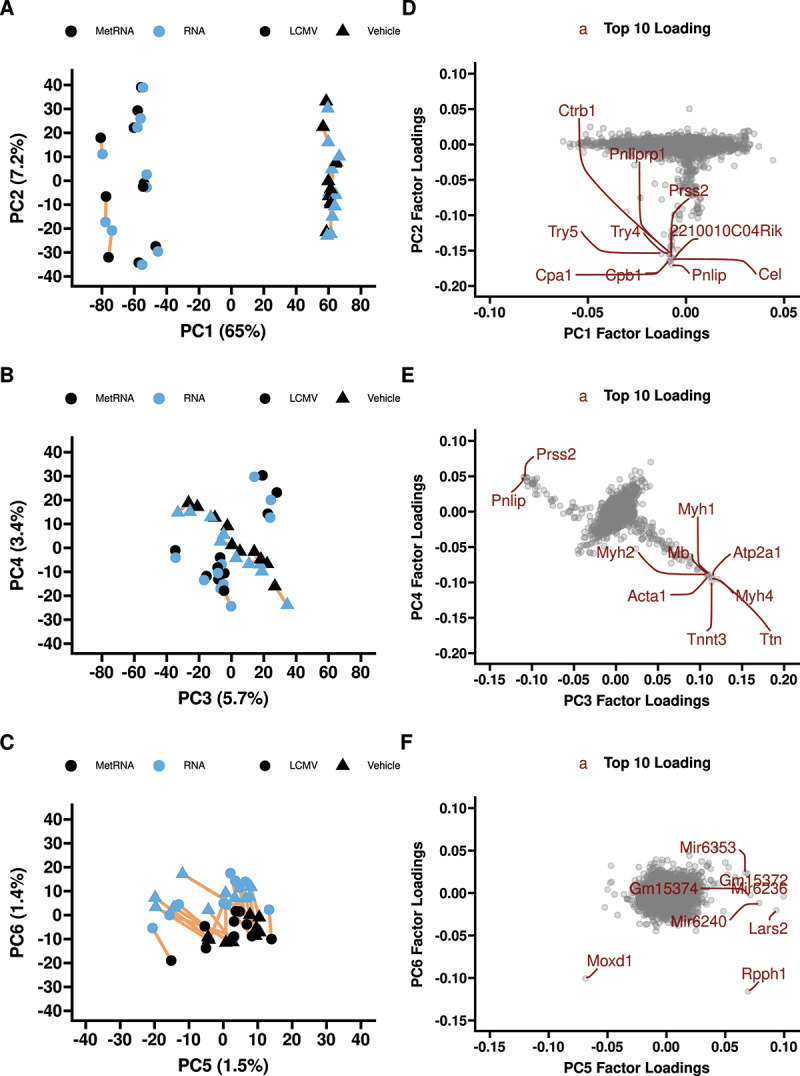
First six principal components of the RNAseq data (A, B, C) with factor loadings (D, E, F). Principal components 7 and beyond explain<1% of the variance each. The top 10 highest contributing genes are labelled in red on the factor loading plots. 65% of the transcriptomic variance is explained by treatment with LCMV and>7% is between subject variance within treatment. PC6 explains~1.4% of the variance and separates extraction methods; PC6 is driven primarily by Rpph1 and Moxd1. *n* = 10 LCMV and *n* = 10 vehicle MetRNA and RNA pairs. Yellow lines connect paired samples (metRNA and RNA from same biological sample).

Principal component analysis revealed a robust separation between LCMV and Veh groups in principal component 1 (PC1, 65% of variance), irrespective of the extraction method, followed by intragroup differences in PC2 (7.2% of variance; Figure 1A). No clusters were apparent in the score plot PCA3 vs PCA4, accounting for 5.7% and 3.4% variance, respectively (Figure 1B). Separation on PCA score plot by extraction method was only apparent in PC5 and PC6 (1.5% and 1.4% of variance, respectively) (Figure 1C). Loading plots for each principal component are shown with the top 10 influential genes highlighted in each (Figure 1D–F). The two genes that were the main contributors to the extraction method separation method in PC6 were *Rpph1* and *Moxd1* (Figure 1F). *Rpph1* was significantly different between the extraction methods in both treatments (Figure S3A), whereas *Moxd1* had no evidence of differing between methods (Figure S3B).

**Figure 2.**
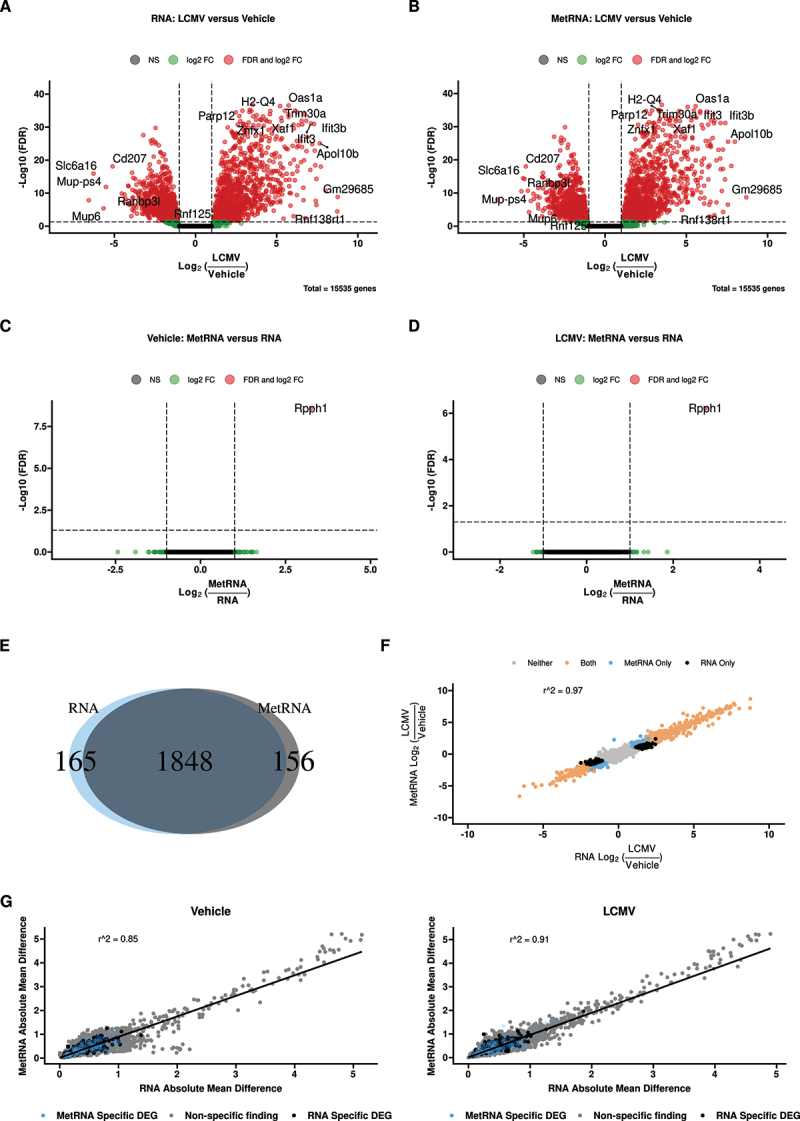
Comparison of the RNAseq data obtained with the two extraction methods (RNA: classical mRNA isolation; MetRNA: metabolite extraction was performed prior to mRNA isolation). (A) and (B) Volcano plots of genes differentially expressed between vehicle and LCMV animals within both extraction methods. (C) and (D) Volcano plots of genes differentially expressed between the extraction methods within vehicle and LCMV animals, respectively. (E) Venn diagram of the genes found to be differentially expressed with FDR<0.05 in both extraction methods, ~85% of the 2,169 genes found to be differentially expressed in at least one extraction method were significantly different in both. (F) Correlation of the log2 fold-change in gene expression (LCMV/Veh) between the two extraction methods. Over 97% of the variance in one extraction method was explained by the other, further genes that were only identified as differentially expressed in one method had only slight changes in estimated fold-change. (G) Correlating mean absolute difference of each gene between the two extraction methods in vehicle and LCMV animals; both were significantly correlated (*p* < 0.05). Genes that were called as differentially expressed in only one of the two extraction methods also have similar dispersion. *n* = 10 pairs per treatment.

We next asked whether prior metabolite extraction affected the detection of significantly differentially expressed genes between biological groups. Volcano plots comparing LCMV/Veh gene expression in RNA (Figure 2A) and MetRNA Figure 2B extraction methods revealed nearly identical distributions with >1,800 differentially expressed genes. In contrast, *Rpph1*, the same transcript identified by principal component analysis (Figure 1E and F), was the only significantly differentially expressed gene by extraction method in both Veh (Figure 2C) and LCMV (Figure 2D) groups. The majority of differentially expressed genes (1,848) between LCMV and Veh were shared between extraction methods (Figure 2E). There were 321 differentially expressed genes that were significant in only one extraction method, with 165 unique to RNA and 156 unique to MetRNA. There was no evidence of gene ontology enrichment on either set of genes (not shown).

A delta–delta comparison of these genes [LCMV_MetRNA – Veh_MetRNA] - [LCMV_RNA – Veh_RNA] produced no significant findings (all FDR >0.999). The median fold-change between genes uniquely significant to an extraction method was 1.04 with 94% of the genes having a log2 (fold change) less than ±1. The probability of obtaining this amount of overlap (>85% shared DEGs) by chance, as estimated via a hypergeometric test, is <0.0001. Further, as seen in Figure 2F and G, the two methods are highly concordant with a Pearson’s R^2^ = 0.97. Taken together, these analyses suggest that alterations in the detection of significantly differentially expressed genes by extraction methods are due to stochastic differences in measurements, rather than a systematic effect of extraction.

### Extraction method does not affect data dispersion

Having demonstrated that prior metabolite extraction does not affect the distribution of differentially expressed genes, we next sought to better understand what effects extraction method has on the global dispersion of RNA. That is, does RNA extraction modality introduce or reduce noise? Within both LCMV and vehicle-injected animals, the absolute mean difference was significantly correlated between MetRNA– and RNA-only transcripts (both *p*-values were <0.001; Figure 2G). Densities estimated on the fold-change in mean absolute differences appeared Gaussian with skewness estimates of only ~ 0.1 for both LCMV and Veh (Figure S4). These low skewness estimates, combined with means approximately equal to the medians, strongly suggest that the changes in dispersion are symmetric about a null difference (i.e. 1-fold difference or log2(fold) = 0) and, therefore, random.

### Integrated pathway analysis

Our data support the conclusion that RNAseq from post-metabolite extraction RNA was at least as informative as traditionally extracted RNA, which enables confident multi-‘omics approaches to understanding metabolic phenotype. To evaluate the capacity of this approach, we interrogated metabolic phenotypes using combined RNAseq and metabolomics from the same sample. As with the broad effects of LCMV on gene expression (Figure 2), we observed that LCMV induced broad changes in the hepatic metabolome (Figure S5). Pathway enrichment analysis of metabolomics data revealed purine, pyrimidine and glycolysis/gluconeogenesis as the most differentially impacted pathways (Figure S6). However, within these pathways, some metabolites were increased and others decreased. This leaves ambiguity as to how these pathways are affected with LCMV.

To gain further insight, we next performed pathway enrichment analysis on RNAseq data. Unsurprisingly, the results of this analysis, as generated by both MetaboAnalyst and the more common GSEA method, were dominated by immune response pathways in LCMV-infected mice (Figure S7). This revealed JAK-STAT signalling pathway, linoleic acid metabolism, beta-Alanine metabolism, neutrophil degranulation, signalling by interleukins and cytokine signalling in the immune system as some of the most impacted pathways. Interestingly, there was little overlap between RNAseq metabolic pathways and those pathways identified with metabolomics data.

**Figure 3.**
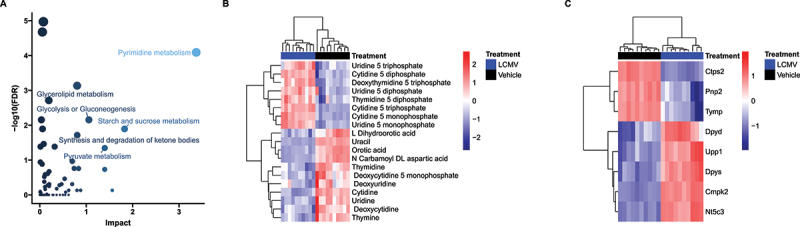
Integrated Metabolic Pathway Enrichment. (A) Plot of how much each pathway examined by MetaboAnalyst was impacted by changes in gene expression and whether or not these changes were significant after FDR adjustment. Data are coloured by impact and sized by FDR values. The pyrimidine metabolism pathway was the most impacted (FDR<0.0001). (B) and (C) Heatmaps of pyrimidine metabolites and transcripts that were measured in this study. For both – omics, we observed both up and down regulation within the pathway and strong clustering within LCMV/Vehicle. *n* = 10 per treatment.

Finally, we integrated metabolite and RNA data and performed a combined pathway analysis Figure 3 and Table 1, revealing pyrimidine metabolism (Kyoto Encyclopedia of Genes and Genomes (KEGG) pathway map 00240) as the most highly impacted pathway (Figure 3A). Pyrimidine metabolism was consistently a significantly and heavily impacted pathway under each of the various centrality measures and weighting methods used for joint integration, often remaining the most affected (Figure S8). Both transcripts and metabolites in the pyrimidine metabolism pathway had bidirectional responses to LCMV (Figure 3B and C), which obscured biological interpretation. First, nucleotides (CTP, CMP, UTP, UDP, UMP, dTTP and dTDP) were decreased in LCMV, whereas metabolites involved in *de novo* pyrimidine synthesis (*N*-carbamoyl-aspartate, dihydroorotate and orotate), nucleosides (cytidine, uridine, thymidine, deoxycytidine, deoxyuridine and thymidine) and nucleobases (uracil, thymine) were increased (Figure 3B and C). When considering metabolomics data in isolation, pyrimidine metabolism is clearly involved in the phenotype. However, is the directionality of the change (i.e. whether *de novo* nucleotide production is elevated to support increased RNA/DNA synthesis or whether nucleotides are being catabolized to produce nucleosides/nucleobases) was unclear.

**Table 1.** Pathway enrichment based on the joint-integration of the transcript and metabolite differences. Only the top five hits are shown. The ‘Total’ column represents the number of compounds and transcripts queried for a given pathway. ‘Expected’: number of hits one would expect in a given pathway by chance. ‘Hits’: the number of differentially expressed genes and differentially abundant metabolites in each pathway. ‘FDR’: Benjamini–Hochberg false discovery rate adjusted p-values for enrichment based on hyper-geometric tests and tight integration of the queries using the betweenness centrality. ‘Impact’: the estimated cumulative impact on the pathway.

	Total	Expected	Hits	Genes	Metabolites	FDR	Impact
Pyrimidine metabolism	99	11	27	8	19	<0.0001	3.4
Starch and sucrose metabolism	37	4.1	11	8	3	0.01	1.8
Pyruvate metabolism	45	5.0	11	6	5	0.045	1.4
Glycolysis or Gluconeogenesis	61	6.8	16	10	6	0.007	1.1
Synthesis and degradation of ketone bodies	10	1.1	5	4	1	0.02	0.81

**Figure 4.**
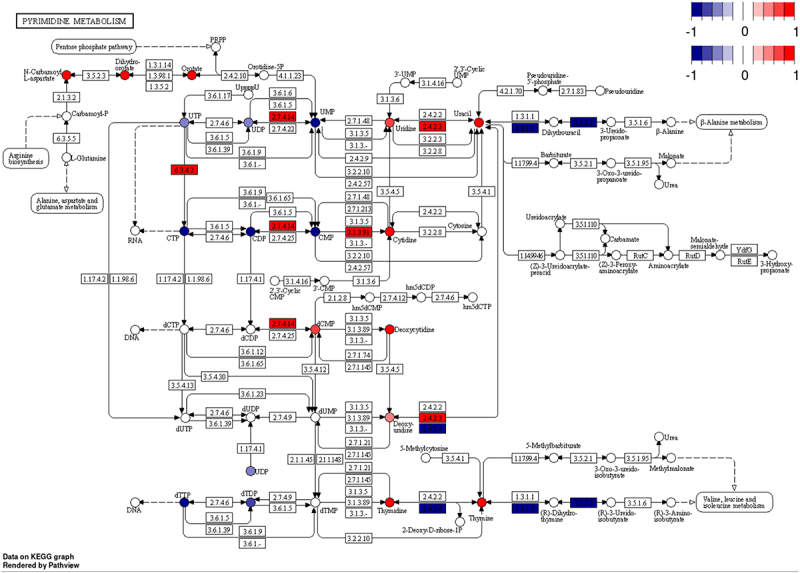
Integrative multi-omics analysis demonstrating biological information gain. Significantly different transcripts (boxes) and metabolites (circles) coloured by log2 fold-change mapped to KEGG pathway.

**Figure 5.**
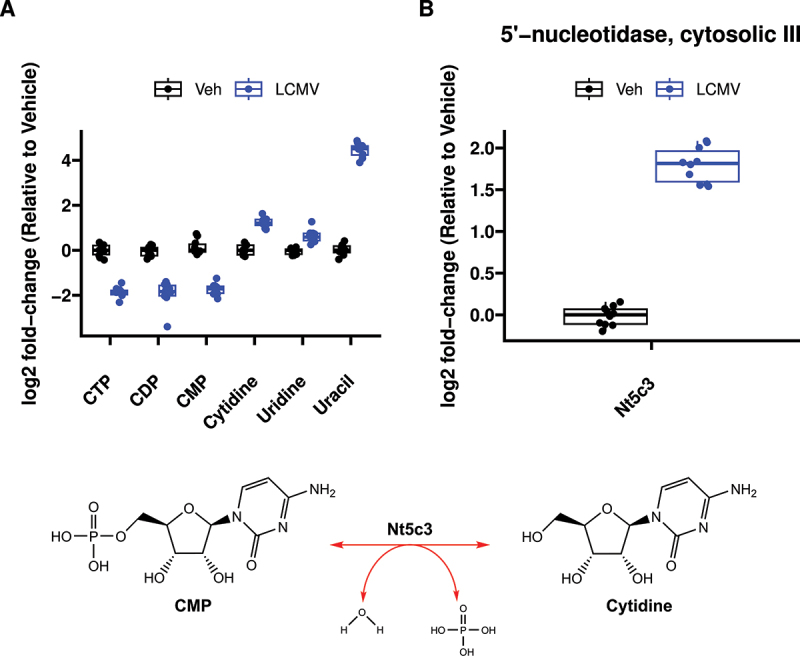
Metabolomic and transcriptomic evidence that cytidine nucleotides are being catabolized to form uracil. (A) Relative abundance of metabolites arranged in biochemical order from CTP → CDP → CMP → Cytidine → Uridine → Uracil. (B) Differential expression of 5’-Nucleotidase, Cytosolic IIIA, the transcript for the enzyme catalysing the CMP → cytidine reaction. *n* = 10 per treatment.

**Figure 6.**
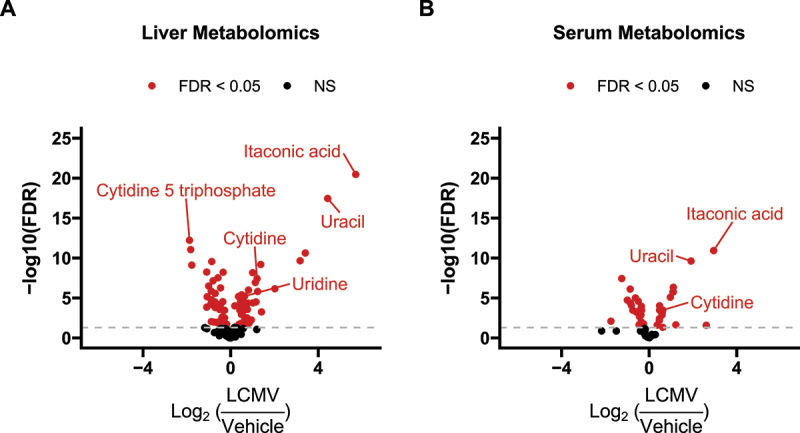
Volcano plots of metabolomics data in the (A) liver and (B) serum reveal strong induction in uracil abundance during LCMV infection. ‘FDR’: Indicates Benjamini-Hochberg false discovery rate adjusted p-values<0.05. ‘NS’: not significant. *n* = 10 per treatment.

To address this, we used gene expression data to inform directionality of the pyrimidine metabolite phenomena. We mapped gene expression and metabolite heatmap signatures onto the KEGG pyrimidine pathway (Figure 4). RNAseq revealed that the expression of genes that encode enzymes catalysing bidirectional nTP ↔ nDP (2.7.4.6), nDP ↔ nMP (2.7.4.14) and nucleoside ↔ nucleobase (2.4.2.3) interconversions were either unchanged or increased. Also, expression of genes encoding proteins that drive dephosphorylation of nucleotide monophosphates to the cognate nucleoside were increased. In contrast, genes encoding enzymes involved in nucleobase catabolism (1.3.1.2 and 3.5.2.2) had decreased expression. To further highlight this pattern, cytidine nucleotides (CTP, CDP and CMP) have decreased abundance (Figure 5A), whereas the gene encoding the cytosolic 5’-nucleotidase 3 (*Nt5c3*) had elevated expression (Figure 5B) and the product of this reaction, cytidine, and its downstream metabolic cognate, uracil, were also elevated (Figure 5A). Interestingly, uracil was among the most elevated metabolites in the liver of LCMV-infected mice (Figure 6A). We further conducted plasma metabolomics on these mice. In line with our observations of increased hepatic uracil content, plasma metabolomics revealed that uracil, along with the inflammatory mediator metabolite itaconate [43,44], were the most strongly induced metabolites in plasma upon LCMV infection (Figure 6B). Together, our data suggest that pyrimidine nucleobases, especially uracil, are being generated in the liver through the catabolism of pyrimidine nucleosides from both *de novo* pyrimidine synthesis and pyrimidine nucleotide catabolism and that they are potentially released into the circulation.

## Discussion

Conducting multiple ‘omics approaches on a single sample provides a more complete picture of the phenotype while avoiding challenges like repeat-sampling and limited quantities, but it also requires the serial extraction of different classes of biomolecules. While numerous such protocols are available [31–37], there is a lack of information on whether and how the readout is affected by additional processing steps. In this study, we used liver tissue from vehicle or acute LCMV-infected mice to assess the effects of prior metabolite extraction on RNAseq data. Our results demonstrate that RNAseq analysis following metabolite extraction yields virtually identical results as traditionally extracted RNA, not only in data dispersion but most importantly in detection of biological effects. Highly correlated variance, abundance and fold-change estimates between extraction methods support that this approach can be broadly applicable to studies where smaller effect sizes are expected. These data provide important and needed validation that co-extraction of metabolites and RNA is a viable approach that is at least as effective as standard approaches that gain a single outcome per sample.

A major advancement of this work is the direct and comprehensive evaluations of RNAseq data quality from RNA that is isolated following metabolite extraction compared to samples only undergoing RNA isolation. We took several steps to allow direct comparison extraction methods rather than sampling variability. First, we used snap-frozen mouse liver tissue and pulverized it into a fine powder in liquid N_2_ to avoid liver regional heterogeneity and allow for repeat sampling with minimal variability. Next, to avoid potential errors induced by extraction efficiency, we fixed the tissue weight to extraction volume ratio for each sample. Finally, we selected the LCMV model system because of its strong biological phenotype, which permitted a comprehensive evaluation of extraction method on a complex biological readout. RNAseq data are commonly interpreted using pathway enrichment analysis, which evaluates the number of significantly differentially expressed genes in each pathway that occur above what would be expected by chance. While this approach can lead to important insight into pathways that may be involved in the phenotype, establishing how that pathway is changing can be challenging. This is especially true for inferring changes in metabolic activity from RNAseq data because metabolic genes can change in response to the metabolite milieu but also be a cellular response to alter metabolism. Similarly, metabolite pool sizes may indicate that a certain pathway is involved, but directionality is unclear. For example, increased abundance of a metabolite could either mean its production is increased or its clearance is decreased. This is further complicated by the biased nature of metabolomics. The wide range of metabolite chemistries and abundances make truly unbiased sampling of the metabolome impossible. Therefore, metabolic pathway involvement is skewed to those in which metabolites are detectable by the analytical method.

Here, we demonstrate that combining RNAseq and metabolite data can lead to a more complete picture of the metabolic phenotype and allow for the generation of actionable hypotheses. Our results support the hypothesis that LCMV infection induces a liver metabolic programme to produce and secrete uracil into circulation. Specifically, levels of CTP, CDP and CMP were found to be decreased in liver extracts upon LCMV infection, while cytidine levels were increased (Figure 5A). These observations are supported by the increased transcript levels of *Nt5c3* (Figure 5B), which encodes for the enzyme that dephosphorylates CMP and UMP to their nucleosides cytidine and uridine, respectively [45]. Furthermore, expression of the enzyme that converts uridine to uracil, uridine phosphorylase (Upp1) [46], is upregulated upon LCMV infection (Figure 3C), and uracil levels are elevated in both liver and serum in LCMV extracts (Figure 6). Thus, while both RNAseq and metabolomics datasets indicated an involvement of pyrimidine metabolism, their integration clearly indicates that pyrimidine nucleotides are being catabolized to support hepatic uracil release. We speculate that a physiological basis for hepatic uracil production during acute infection may be to supplement activated and proliferating immune cells with nucleotides imperative for RNA synthesis. Further investigation is necessary to test this model, but this example highlights the advantages of integrating multiple ‘omics data to generate an actionable hypothesis.

In our multi-‘omic pathway analysis, we focused on integrating the metabolome and transcriptome data using MetaboAnalyst’s tight integration of the queries and betweenness centrality. Under this integration method, the multi-omics results are pooled into a single query where each hit has the same weight. With 36 times more differentially expressed genes than differentially abundant metabolites, this tight integration can result in transcriptomic differences dominating the results. We found strong metabolite evidence of pyrimidine metabolism being impacted in this study. If there were no transcript evidence, we would not expect pyrimidine metabolism to remain one of the top hits in the joint analysis. However, since we did observe differences in the regulation of genes related to pyrimidine metabolism, the result was conserved. This yielded higher confidence that the pyrimidine pathway was truly impacted. The betweenness centrality identifies compounds/transcripts that act as ‘brokers’ or ‘bridges’ between subunits of a pathway. In the context of pyrimidine metabolism, when compounds/transcripts with high betweenness centrality are altered, it indicates that the entire cascade of biochemical signalling has been fundamentally altered. For completeness, we explored other centrality measures and weighted integration methods, each of which indicated pyrimidine metabolism as a significantly impacted pathway (Figure S6). The concordance of these measures provides confidence that infection with LCMV results in a strong involvement in pyrimidine metabolism.

As pyrimidine metabolism was the most significantly impacted pathway in our multi-‘omic analysis, we used it as an example to highlight the potential of multi-‘omic integration. However, it is important to note that other metabolic pathways were also significantly affected. Joint-integration methods revealed other candidate pathways to explore, notably ‘Starch and Sucrose Metabolism’ and ‘Degradation of Ketone Bodies’, which were only identified as being highly impacted in the joint analysis.

A general limitation of metabolomics is incomplete coverage of the metabolome. While RNAseq data can provide a comprehensive view of transcripts above a limit of detection threshold, metabolomics coverage is dependent on both analytical methods (metabolite extraction technique, chromatography, ionization type, polarity, and instrument sensitivity) and biological abundance. Therefore, it is important to consider that metabolomics studies, and subsequent multi-omics integration, will be biased towards the metabolites that are detectable in the method. In this light, metabolomics can be used positively, for example, to provide orthogonal validation of RNAseq results or highlight altered metabolic pathways independent of transcription. However, the use of metabolomics data to rule out certain metabolic pathways is not recommended because metabolic pathways are often incompletely covered or simply not detected. By extension, RNAseq results may be used for direct follow-up metabolomics analyses that target specific pathways/compounds of interest.

The current study presents an in-depth evaluation of the effects of prior metabolite extraction on mRNA quality, transcriptomic coverage and phenotypic insight. However, there are limitations to our approach that warrant discussion. First, whether extraction modality affects non-protein coding RNAs was not assessed. Our analysis revealed that *Rpph1* was the only transcript affected by the extraction method. *Rpph1* is a highly conserved long non-coding RNA (lncRNA) that encodes the RNA component of RNaseP. In eukaryotes, RNaseP exists as a riboenzyme with up to 10 protein subunits [47,48]. Although it is not polyadenylated as mRNA, *Rpph1* does appear in other mRNA-sequencing datasets [49]. In the light of our observations that MetRNA improves total RNA yield (Figure S1), we posit that the observed increase in *Rpph1* expression in metRNA may indicate that the additional steps prior to RNA extraction improved the extraction of protein-complexed RNAs. Whether metabolite extraction similarly boosts the recovery of other lncRNAs was not evaluated here, but our data encourage further investigations in this area. Secondly, liver tissue was used as a model system due to its size (to enable multiple sampling) and its strong phenotypic response to LCMV infection. It is unknown whether RNA in tissues with different compositions, for example, high lipid content in adipose tissue, will respond the same. Finally, the tissue-to-solvent ratio was carefully controlled to avoid the potentially confounding influence of extraction efficiency, and only a single commonly used metabolite extraction modality (80% methanol) was used. These factors should be considered when designing a study to co-extract metabolites and RNA.

## Conclusion

Often biological material is limited, and the ability to maximize information gained from the same sample is beneficial. Even when tissue is in abundance, regional heterogeneity within a tissue can limit the utility of multi-omics approaches. Our study demonstrates that sequentially extracting metabolites and RNA for metabolomics and transcriptomic analyses does not fundamentally impact gene transcript abundance. We also show that by jointly integrating changes to both omics’ datasets through enrichment analyses, we can identify metabolic phenotypes with higher statistical confidence, while gaining additional biological insight into how pathways are altered. Finally, through the implementation of these methods, we reveal a novel finding that infecting mice with LCMV results in significant and dramatic changes to pyrimidine metabolism. This work provides validation that sequential extraction of metabolites and RNA from a single sample can be confidently used for multi-omics analysis.

## Supplementary Material

Supplemental Material

## References

[1] Zhu J, Thompson CB. Metabolic regulation of cell growth and proliferation. Nat Rev Mol Cell Biol. 2019;20(7):436–450. 30976106 10.1038/s41580-019-0123-5 PMC6592760

[2] Dai Z, Ramesh V, Locasale JW. The evolving metabolic landscape of chromatin biology and epigenetics. Nat Rev Genet. 2020;21(12):737–753. 32908249 10.1038/s41576-020-0270-8 PMC8059378

[3] Wang YP, Li JT, Qu J, et al. Metabolite sensing and signaling in cancer. J Biol Chem. 2020;295(33):11938–11946. DOI:10.1074/jbc.REV119.007624 32641495 PMC7450124

[4] Pavlova NN, Zhu J, Thompson CB. The hallmarks of cancer metabolism: still emerging. Cell Metab. 2022;34(3):355–377. 35123658 10.1016/j.cmet.2022.01.007 PMC8891094

[5] Inigo M, Deja S, Burgess SC. Ins and outs of the TCA cycle: the central role of anaplerosis. Annu Rev Nutr. 2021;41(1):19–47. 34270333 10.1146/annurev-nutr-120420-025558

[6] Martinez-Reyes I, Chandel NS. Mitochondrial TCA cycle metabolites control physiology and disease. Nat Commun. 2020;11(1):102. 31900386 10.1038/s41467-019-13668-3 PMC6941980

[7] Loaiza A, Porras OH, Barros LF. Glutamate triggers rapid glucose transport stimulation in astrocytes as evidenced by real-time confocal microscopy. J Neurosci. 2003;23(19):7337–7342. 12917367 10.1523/JNEUROSCI.23-19-07337.2003 PMC6740433

[8] Khemtong C, Carpenter NR, Lumata LL, et al. Hyperpolarized 13C NMR detects rapid drug-induced changes in cardiac metabolism. Magn Reson Med. 2015;74(2):312–319. DOI:10.1002/mrm.25419 25168480 PMC4344937

[9] Golman K, ‘t Zandt R, Thaning M. Real-time metabolic imaging. Proc Natl Acad Sci U S A. 2006;103(30):11270–11275. 16837573 10.1073/pnas.0601319103 PMC1544077

[10] Rauckhorst AJ, Borcherding N, Pape DJ, et al. Mouse tissue harvest-induced hypoxia rapidly alters the in vivo metabolome, between-genotype metabolite level differences, and (13)C-tracing enrichments. Mol Metab. 2022;66:101596. 36100179 10.1016/j.molmet.2022.101596 PMC9589196

[11] Shamir M, Bar-On Y, Phillips R, et al. SnapShot: timescales in cell biology. Cell. 2016;164:1302. 26967295 10.1016/j.cell.2016.02.058

[12] Yin X, Bose D, Kwon A, et al. Integrating transcriptomics, metabolomics, and GWAS helps reveal molecular mechanisms for metabolite levels and disease risk. Am J Hum Genet. 2022;109(10):1727–1741. DOI:10.1016/j.ajhg.2022.08.007 36055244 PMC9606383

[13] Cheng S, Shah SH, Corwin EJ, et al. PotentiaL impact and study considerations of metabolomics in cardiovascular health and disease: a scientific statement from the American heart association. Circ Cardiovasc Genet. 2017;10(2):10. DOI:10.1161/HCG.0000000000000032 PMC6001363 28360086

[14] Yu B, Zheng Y, Alexander D, et al. Genetic determinants influencing human serum metabolome among African Americans. PLoS Genet. 2014;10(3):e1004212. DOI:10.1371/journal.pgen.1004212 24625756 PMC3952826

[15] Zhou S, Morgante F, Geisz MS, et al. Systems genetics of the drosophila metabolome. Genome Res. 2020;30(3):392–405. DOI:10.1101/gr.243030.118 31694867 PMC7111526

[16] Garcia-Canaveras JC, Chen L, Rabinowitz JD. The tumor metabolic microenvironment: lessons from lactate. Cancer Res. 2019;79(13):3155–3162. 31171526 10.1158/0008-5472.CAN-18-3726 PMC6606343

[17] Kaymak I, Williams KS, Cantor JR, et al. Immunometabolic interplay in the tumor microenvironment. Cancer Cell. 2021;39:28–37. 33125860 10.1016/j.ccell.2020.09.004 PMC7837268

[18] Martinez-Reyes I, Chandel NS. Cancer metabolism: looking forward. Nat Rev Cancer. 2021;21(10):669–680. 34272515 10.1038/s41568-021-00378-6

[19] Geiszler PC, Ugun-Klusek A, Lawler K, et al. Dynamic metabolic patterns tracking neurodegeneration and gliosis following 26S proteasome dysfunction in mouse forebrain neurons. Sci Rep. 2018;8(1):4833. DOI:10.1038/s41598-018-23155-2 29555943 PMC5859111

[20] Ryu WI, Bormann MK, Shen M, et al. Brain cells derived from Alzheimer’s disease patients have multiple specific innate abnormalities in energy metabolism. Mol Psychiatry. 2021;26(10):5702–5714. DOI:10.1038/s41380-021-01068-3 33863993 PMC8758493

[21] Huo Z, Yu L, Yang J, et al. Brain and blood metabolome for Alzheimer’s dementia: findings from a targeted metabolomics analysis. Neurobiol Aging. 2020;86:123–133. 31785839 10.1016/j.neurobiolaging.2019.10.014 PMC6995427

[22] Troha K, Ayres JS. Metabolic adaptations to infections at the organismal level. Trends Immunol. 2020;41:113–125. 31959515 10.1016/j.it.2019.12.001 PMC7409656

[23] Ayres JS. Immunometabolism of infections. Nat Rev Immunol. 2020;20(2):79–80. 31892735 10.1038/s41577-019-0266-9

[24] Beisel WR. Metabolic response to infection. Annu Rev Med. 1975;26(1):9–20. 1096783 10.1146/annurev.me.26.020175.000301

[25] Palmer CS. Innate metabolic responses against viral infections. Nat Metab. 2022;4(10):1245–1259. 36266542 10.1038/s42255-022-00652-3

[26] Wishart DS. Metabolomics for investigating physiological and pathophysiological processes. Physiol Rev. 2019;99(4):1819–1875. 31434538 10.1152/physrev.00035.2018

[27] Jang C, Chen L, Rabinowitz JD. Metabolomics and Isotope Tracing. Cell. 2018;173:822–837. 29727671 10.1016/j.cell.2018.03.055 PMC6034115

[28] Kaymak I, Luda KM, Duimstra LR, et al. Carbon source availability drives nutrient utilization in CD8+ T cells. Cell Metab. 2022;34(9):1298–311 e6. DOI:10.1016/j.cmet.2022.07.012 35981545 PMC10068808

[29] Maharjan RP, Ferenci T. Global metabolite analysis: the influence of extraction methodology on metabolome profiles of Escherichia coli. Anal Biochem. 2003;313:145–154. 12576070 10.1016/s0003-2697(02)00536-5

[30] Dettmer K, Nurnberger N, Kaspar H, et al. Metabolite extraction from adherently growing mammalian cells for metabolomics studies: optimization of harvesting and extraction protocols. Anal Bioanal Chem. 2011;399:1127–1139. 21125262 10.1007/s00216-010-4425-x

[31] Leuthold P, Schwab M, Hofmann U, et al. SimultaneouS extraction of RNA and metabolites from single kidney tissue specimens for combined transcriptomic and metabolomic profiling. J Proteome Res. 2018;17:3039–3049. 30091608 10.1021/acs.jproteome.8b00199

[32] Valledor L, Escandon M, Meijon M, et al. A universal protocol for the combined isolation of metabolites, DNA, long RNAs, small RNAs, and proteins from plants and microorganisms. Plant J. 2014;79:173–180. 24804825 10.1111/tpj.12546

[33] Roume H, Heintz-Buschart A, Muller EE, et al. Sequential isolation of metabolites, RNA, DNA, and proteins from the same unique sample. Methods Enzymol. 2013;531:219–236. 24060123 10.1016/B978-0-12-407863-5.00011-3

[34] Kang J, David L, Li Y, et al. Three-in-one simultaneous extraction of proteins, metabolites and lipids for multi-omics. Front Genet. 2021;12:635971. 33936167 10.3389/fgene.2021.635971 PMC8082496

[35] Salem M, Bernach M, Bajdzienko K, et al. A simple fractionated extraction method for the comprehensive analysis of metabolites, lipids, and proteins from a single sample. J Vis Exp. 2017. DOI:10.3791/55802 PMC5608179 28605387

[36] Nicora CD, Sims AC, Bloodsworth KJ, et al. Metabolite, Protein, and Lipid Extraction (MPLEx): a method that simultaneously inactivates middle east respiratory syndrome coronavirus and allows analysis of multiple host cell components following infection. Methods Mol Biol. 2020;2099:173–194. 31883096 10.1007/978-1-0716-0211-9_14 PMC7121680

[37] Sapcariu SC, Kanashova T, Weindl D, et al. Simultaneous extraction of proteins and metabolites from cells in culture. MethodsX. 2014;1:74–80. 26150938 10.1016/j.mex.2014.07.002 PMC4472845

[38] Cheng ML, Nakib D, Perciani CT, et al. The immune niche of the liver. Clin Sci (Lond). 2021;135:2445–2466. 34709406 10.1042/CS20190654

[39] Wherry EJ, Blattman JN, Murali-Krishna K, et al. Viral persistence alters CD8 T-cell immunodominance and tissue distribution and results in distinct stages of functional impairment. J Virol. 2003;77:4911–4927. 12663797 10.1128/JVI.77.8.4911-4927.2003 PMC152117

[40] Badovinac VP, Porter BB, Harty JT. Programmed contraction of CD8(+) T cells after infection. Nat Immunol. 2002;3:619–626. 12055624 10.1038/ni804

[41] Pang Z, Zhou G, Ewald J, et al. Using MetaboAnalyst 5.0 for LC-HRMS spectra processing, multi-omics integration and covariate adjustment of global metabolomics data. Nat Protoc. 2022;17:1735–1761. 35715522 10.1038/s41596-022-00710-w

[42] Wu T, Hu E, Xu S, et al. clusterProfiler 4.0: a universal enrichment tool for interpreting omics data. Innovation (Camb). 2021;2:100141. 34557778 10.1016/j.xinn.2021.100141 PMC8454663

[43] Lin J, Ren J, Gao DS, et al. The Emerging Application of Itaconate: promising Molecular Targets and Therapeutic Opportunities. Front Chem. 2021;9:669308. 34055739 10.3389/fchem.2021.669308 PMC8149739

[44] Mills EL, Ryan DG, Prag HA, et al. Itaconate is an anti-inflammatory metabolite that activates Nrf2 via alkylation of KEAP1. Nature. 2018;556:113–117. 29590092 10.1038/nature25986 PMC6047741

[45] Amici A, Emanuelli M, Magni G, et al. Pyrimidine nucleotidases from human erythrocyte possess phosphotransferase activities specific for pyrimidine nucleotides. FEBS Lett. 1997;419:263–267. 9428647 10.1016/s0014-5793(97)01464-6

[46] Watanabe S, Uchida T. Cloning and expression of human uridine phosphorylase. Biochem Biophys Res Commun. 1995;216(1):265–272. 7488099 10.1006/bbrc.1995.2619

[47] Baer M, Nilsen TW, Costigan C, et al. Structure and transcription of a human gene for H1 RNA, the RNA component of human RNase P. Nucleic Acids Res. 1990;18:97–103. 2308839 10.1093/nar/18.1.97 PMC330208

[48] Chamberlain JR, Lee Y, Lane WS, et al. Purification and characterization of the nuclear RNase P holoenzyme complex reveals extensive subunit overlap with RNase MRP. Genes Dev. 1998;12:1678–1690. 9620854 10.1101/gad.12.11.1678 PMC316871

[49] Zhang P, Sun Y, Peng R, et al. Long non-coding RNA Rpph1 promotes inflammation and proliferation of mesangial cells in diabetic nephropathy via an interaction with Gal-3. Cell Death Dis. 2019;10:526. 31285427 10.1038/s41419-019-1765-0 PMC6614467

