# Supplementary material for: Prior metabolite extraction fully preserves RNAseq quality and enables integrative multi-‘omics analysis of the liver metabolic response to viral infection
Source: RNA Biol. 2023 Apr 24;20(1):186–97. doi: 10.1080/15476286.2023.2204586 (PMC10132226; doi:10.1080/15476286.2023.2204586)
Supplement: Supplemental Material [file KRNB_A_2204586_SM3544.zip › 20230309_Supplemental Material_R01_final.docx]

**Supplementary Materials for:**

Prior metabolite extraction fully preserves RNAseq quality and enables integrative multi-‘omics analysis of the liver metabolic response to viral infection

**Authors:**

Zachary B Madaj^1,5^, Michael S. Dahabieh^2^, Vijayvardhan Kamalumpundi^4,5^, Brejnev Muhire^2^, Dean J. Pettinga^1,5^, Rebecca A. Siwicki^3,5^, Abigail E. Ellis^4,5^, Christine Isaguirre^4,5^, Martha L. Escobar Galvis^5^, Lisa DeCamp^2^, Russell G. Jones^2^, Scott A. Givan^1,5^, Marie Adams^3,5^, Ryan D. Sheldon^4,5†^

**Institutional Affiliations:**

^1^Bioinformatics and Biostatistics Core, ^2^Department of Metabolic and Nutritional Programming, ^3^Genomics Core, ^4^Mass Spectrometry Core, ^5^Core Technologies and Services, Van Andel Institute, Grand Rapids, MI, USA. ^†^Corresponding Author

**Corresponding Author:**

Ryan D. Sheldon, PhD

Van Andel Research Institute

333 Bostwick Ave. NE, Grand Rapids, MI 49503

+1-616-234-5727

[ryan.sheldon@vai.org](mailto:ryan.sheldon@vai.org)


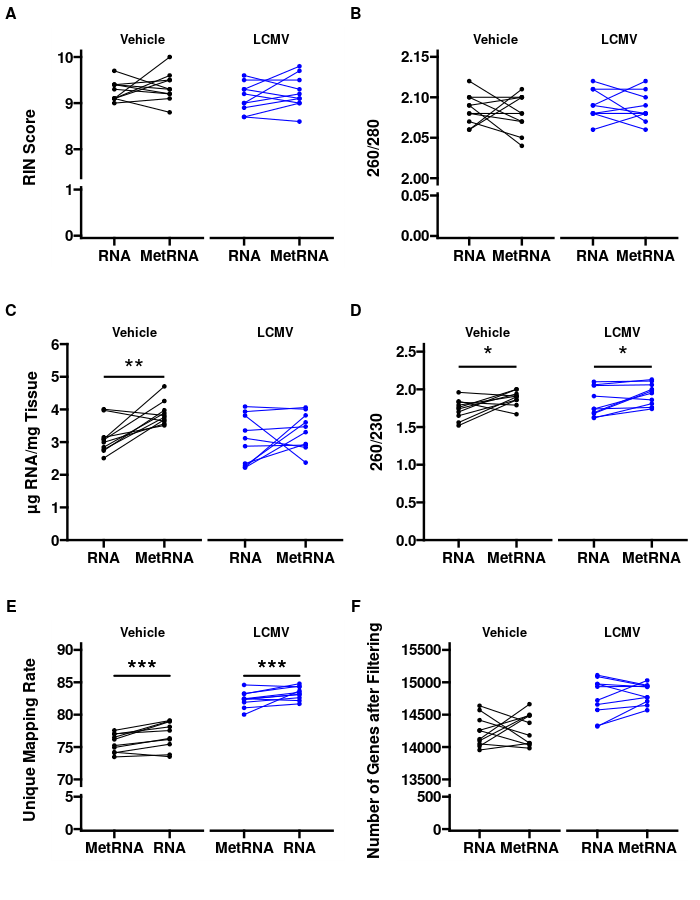


Figure S1. Characterization of the quality and yield of the mRNA after extraction. A-D) RIN score, 260/280, µg RNA/mg tissue, and 260/230 were analyzed via robust linear mixed-effects models with a random intercept for each individual mouse to account for MetRNA and RNA pairs. E) The unique mapping rate was analyzed via a beta mixed-effects model with random intercepts for each mouse pair. F) The number of genes detected after filtering was analyzed using a negative binomial mixed-effects model with a random intercept for each mouse pair. All models included a *treatment x extraction* method interaction in the main effects. ‘*’ p < 0.05, ‘**’ p <0.01, ‘***’ p <0.001.


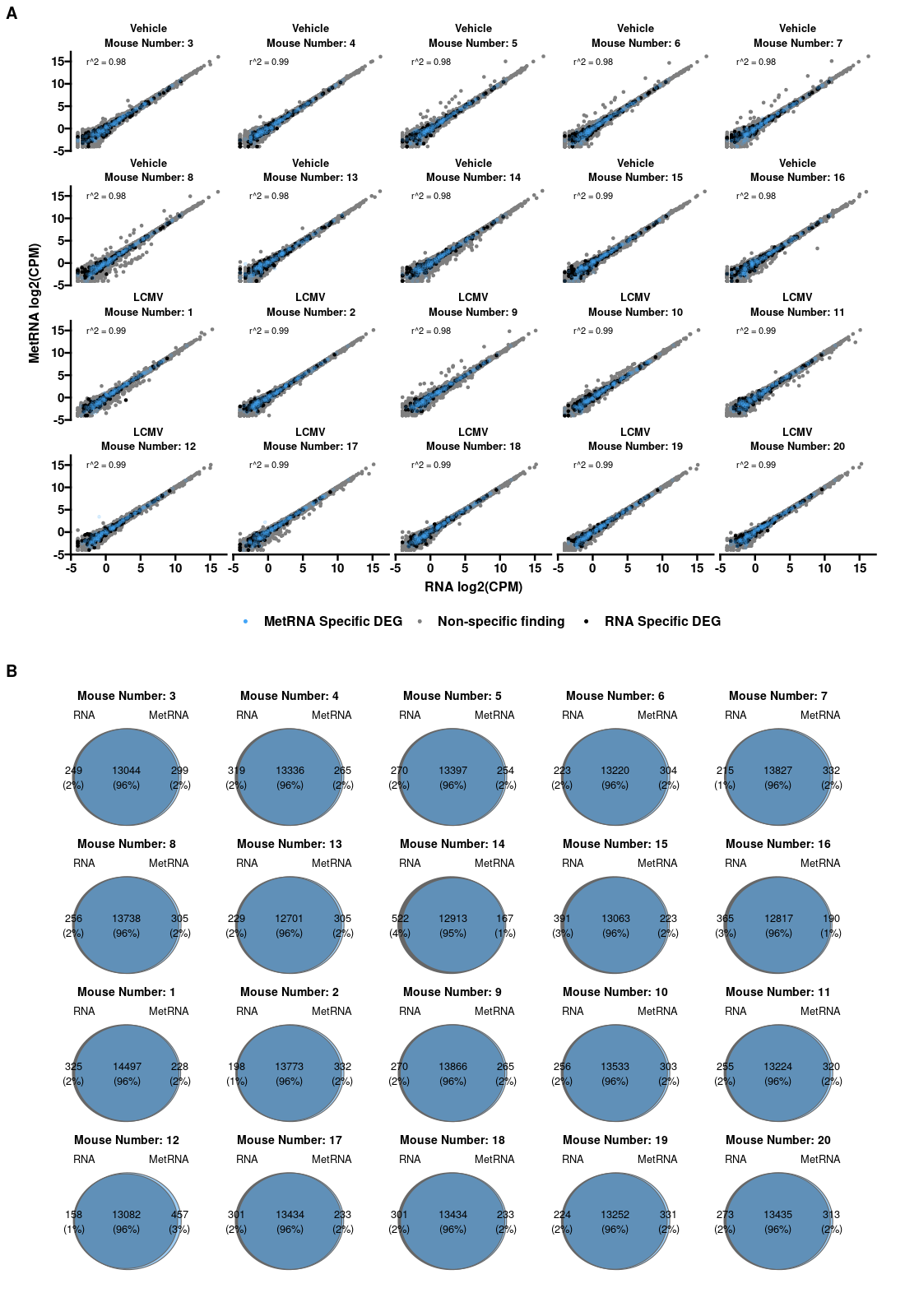


Figure S2. A) Correlating normalized gene expression within each individual animal. *R^2^* :Pearson’s correlation coefficient, squared. B) Overlap in genes detected after filtering low counts (i.e. genes with raw counts >10).


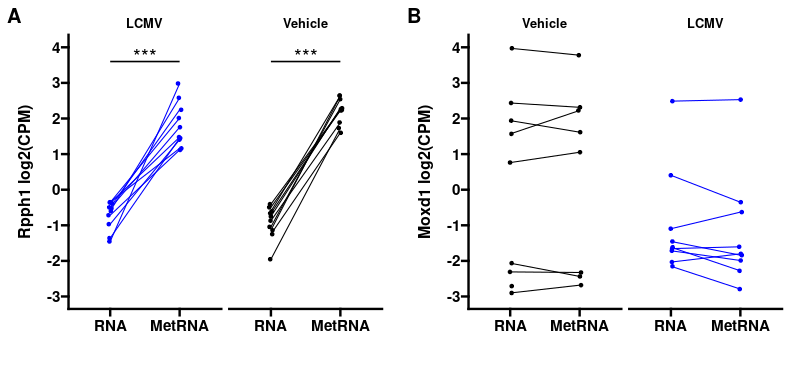


Figure S3. Plots of log2 normalized Rpph1 and Moxd1 counts, the top two contributors to PC6.- ‘*’ FDR p < 0.05, ‘**’ FDR p <0.01, ‘***’ FDR p <0.001. Genes were analyzed via edgeR using the workflow described in the methods section.


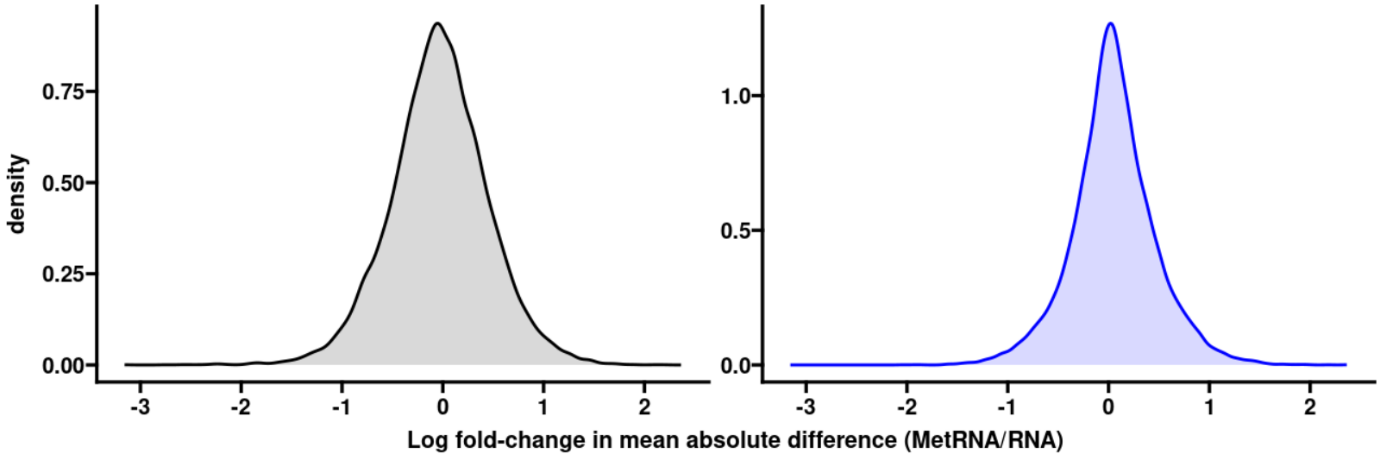


Figure S4. Density of log2 fold-change in the mean absolute difference of each gene between MetRNA and RNA. log2(mean absolute difference MetRNA/ mean absolute difference RNA)


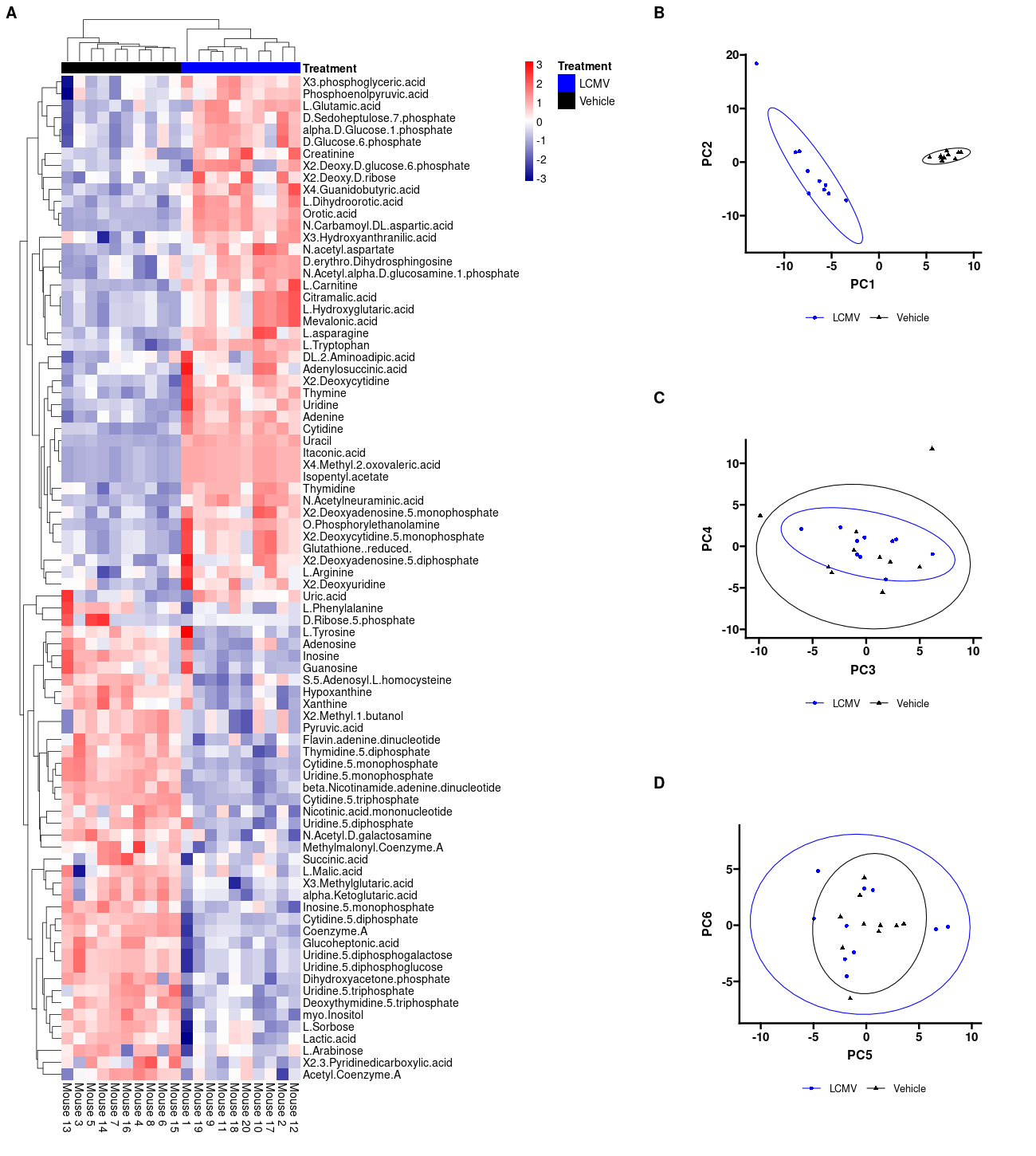
Figure S5. Liver metabolomics analysis. Differentially abundant metabolites show strong clustering by group (LCMV vs Veh) in both a heatmap and the first 6 principal components plotted in pairs.


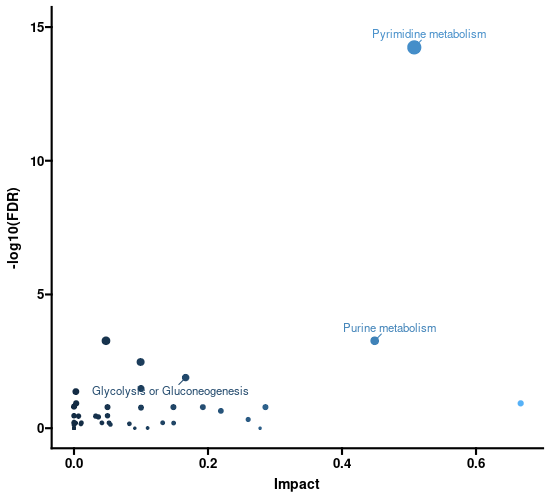


|  | **Total** | **Expected** | **Hits** | **FDR** | **Impact** |
| --- | --- | --- | --- | --- | --- |
| Pyrimidine metabolism | 39 | 1.8 | 19 | <0.0001 | 0.51 |
| Purine metabolism | 66 | 3.0 | 12 | 0.0005 | 0.45 |
| Glycolysis or Gluconeogenesis | 26 | 1.2 | 6 | 0.013 | 0.17 |
| Pyruvate metabolism | 22 | 1.0 | 5 | 0.033 | 0.1 |
| Citrate cycle (TCA cycle) | 20 | 0.90 | 6 | 0.003 | 0.1 |

Figure S6. Metabolite-only pathway analysis.


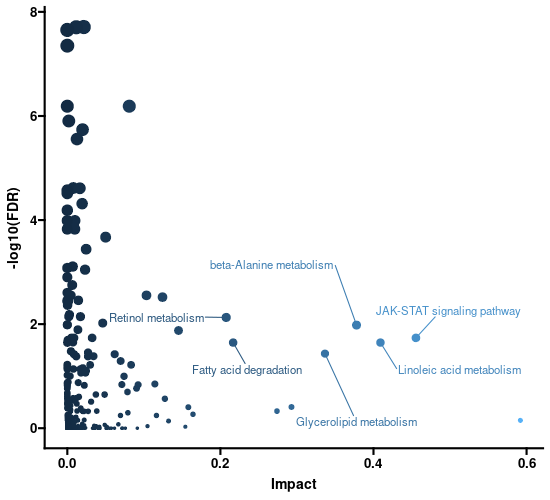

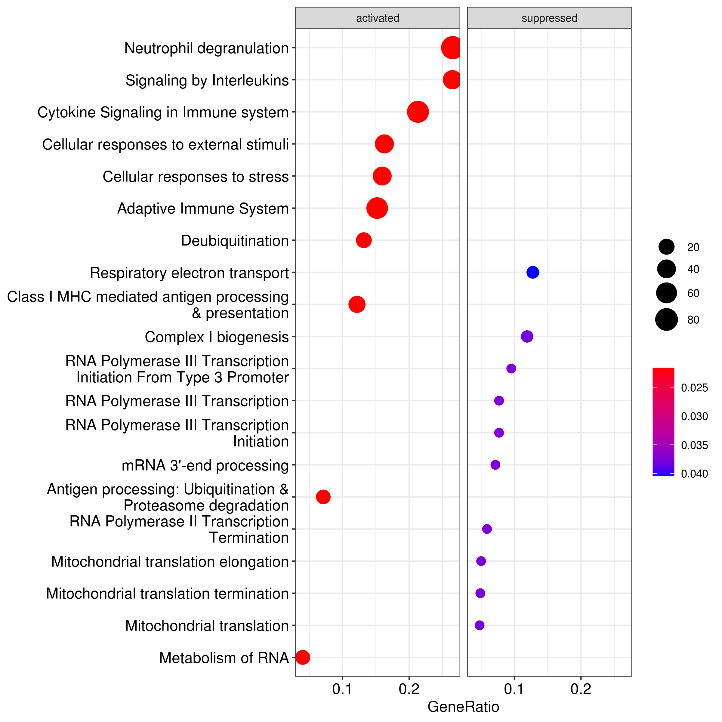


|  | **Total** | **Expected** | **Hits** | **FDR** | **Impact** |
| --- | --- | --- | --- | --- | --- |
| JAK-STAT signaling pathway | 165 | 14.6 | 26 | 0.02 | 0.46 |
| Linoleic acid metabolism | 50 | 4.4 | 11 | 0.02 | 0.41 |
| beta-Alanine metabolism | 32 | 2.8 | 9 | 0.01 | 0.38 |
| Glycerolipid metabolism | 61 | 5.4 | 12 | 0.04 | 0.34 |
| Fatty acid degradation | 50 | 4.4 | 11 | 0.02 | 0.22 |

Figure S7. Gene-only pathway analysis and pathway enrichment from clusterProfiler.


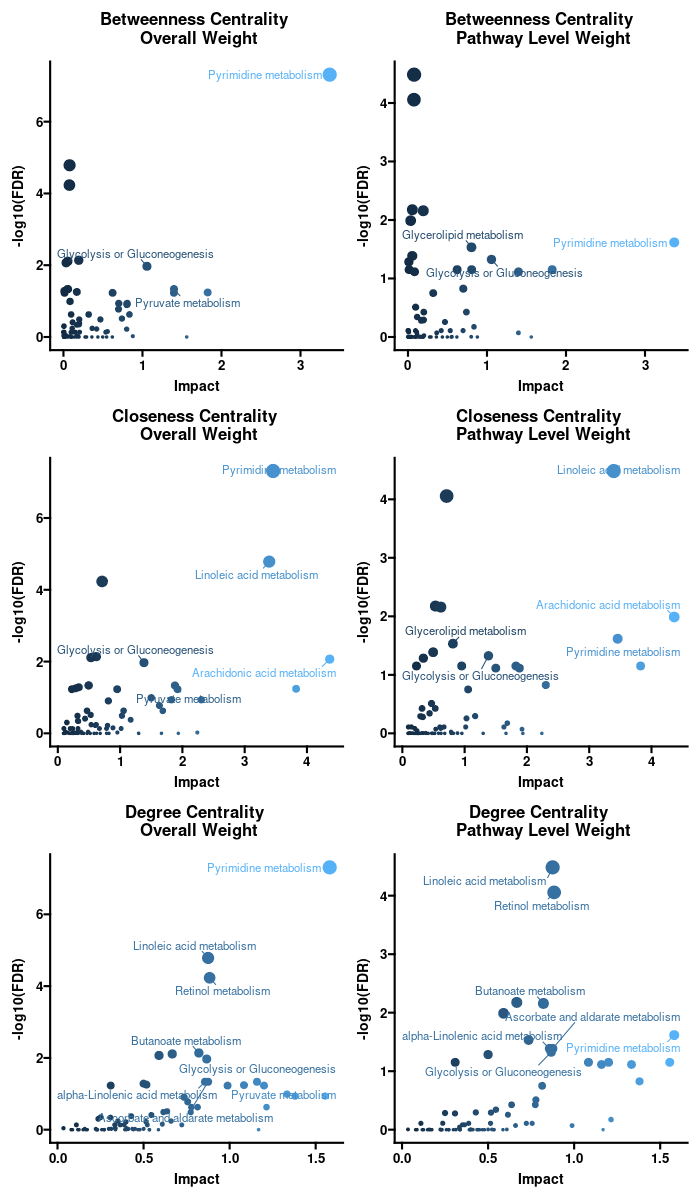


Figure S8. Joint metabolic and transcriptomic enrichment analysis under other degree centralities and weighting methods.
